# Supplementary material for: EuDockScore: Euclidean graph neural networks for scoring protein–protein interfaces
Source: Bioinformatics. 2024 Oct 23;40(11):btae636. doi: 10.1093/bioinformatics/btae636 (PMC11543620; doi:10.1093/bioinformatics/btae636)
Supplement: btae636_Supplementary_Data [file btae636_supplementary_data.pdf]

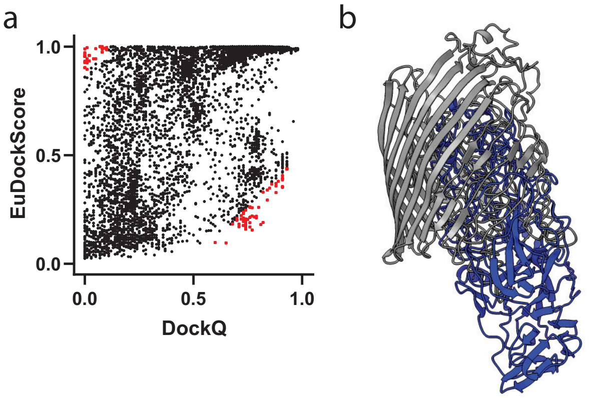

Fig. S1: (a) Comparison of DockQ and EuDockScore for dimers in the CASP15 competition. Outliers are identified using the Interquartile Range (IQR) method and highlighted in red. (b) An example structure of an outlier. The PDB file is `H1129TS245_2.pdb`. Each chain in the dimer is colored grey and blue, respectively.
